# An Update on the Effect of Physical Exercise on Depressive Disorder: A Systematic Review with Meta-Analysis and Meta-Regression of Randomized Controlled Trials

**DOI:** 10.3390/jfmk11010009

**Published:** 2025-12-25

**Authors:** Arnulfo Ramos-Jiménez, Rosa P. Hernández-Torres, Javier A. Ramos-Hernández, Marina Trejo-Trejo, Isaac A. Chávez-Guevara

**Affiliations:** 1 Department of Health Sciences, Biomedical Sciences Institute, Ciudad Juárez Autonomous University, Ciudad Juárez 32315, Mexico; 2 Faculty of Physical Culture Sciences, Autonomous University of Chihuahua, Ciudad Juárez 32310, Mexico; rhernant@uach.mx; 3 Faculty of Medicine, Autonomous University of Nuevo Leon, University Campus, Monterrey 64460, Mexico; javier.ramoshrn@uanl.edu.mx; 4 Faculty of Sports, Universidad Autónoma de Baja California, Campus Mexicali, Mexicali 21100, Mexico; marina.trejo@uabc.edu.mx; 5 Faculty of Sports, Universidad Autónoma de Baja California, Campus Ensenada, Ensenada 22860, Mexico; isaac.chavez.guevara@uabc.edu.mx

**Keywords:** depression, major depressive disorder, psychological disease, Bayesian meta-analyses, health care, alternative therapy, exercise intensity, supervised physical exercise, Hamilton Depression Rating Scale, Beck Depression Inventory

## Abstract

**Background**: Physical exercise (PE) has emerged as a promising intervention for depressive disorder (DD), yet its efficacy and optimal implementation remain under investigation. Objective: To thoroughly assess the effectiveness of supervised PE as a conventional intervention for adults with DD. **Methods**: A comprehensive literature search was conducted across PubMed/MEDLINE, EBSCOhost, Ovid, Web of Science, and Scopus. Randomized controlled trials (RCTs) published between 2010–2025 involving adults with DD without other comorbidities under supervised exercise interventions were selected. Methodological rigor was ensured through two independent reviewers and adherence to PRISMA 2020 guidelines. The influence of moderating variables [total work performed (workload) and the instrument used to evaluate DD (instrument)] was analyzed using meta-regression. The pooled effect size was estimated using both frequentist and Bayesian meta-analyses. **Results**: From 15,542 screened records, 20 RCTs were selected. Workload and instrument account for 60% and 15% of the variance in the effect size, respectively. Both frequentist and Bayesian meta-analyses showed that supervised PE reduces depressive symptoms (standardized mean difference = 0.82; 95% CI: 0.54–1.11; I^2^ = 76%, and 0.61; 95% CI: −0.06–0.95; I^2^ = 51, respectively). **Conclusions**: PE could be a valuable complementary intervention to reduce depressive symptoms in adults with depression. PROSPERO (CRD420251121919).

## 1. Introduction

Depressive disorder represents a significant challenge to global public health, affecting millions of individuals worldwide and imposing considerable societal and personal burdens [1]. With an estimated prevalence of 332 million people globally, major depressive disorder (MDD) is a primary cause of disability, with projections indicating an even greater future burden [2]. Data from 2021 in the United States indicate that approximately 21.0 million adults (8.3%) experienced at least one episode of MDD, with a higher prevalence among females (10.3%) than among males (6.3%) [3]. Symptoms encompass persistent low mood, anhedonia, fatigue, low self-esteem, and hopelessness [1]. Moreover, comorbidities such as substance abuse, suicidal ideation, and chronic pain are also prevalent [4]. These conditions diminish quality of life and impose substantial economic costs due to decreased productivity. Depression is not merely a mental health disorder but also a major risk factor for numerous adverse health outcomes, including higher mortality rates, chronic diseases, disability, and poor prognosis in related conditions [5]. Furthermore, a complex interplay between biological, psychological, and social factors elevates the risk of depression, including sleep disturbances, trauma, low social support, and socioeconomic disadvantages [6].

The pursuit of effective, accessible, and sustainable treatments for depressive disorder is thus an urgent priority. Especially, numerous patients with MDD do not achieve remission or discontinue treatment due to various factors, including illness severity, comorbidities, high patient expectations, and adverse medication side effects [7]. Consequently, over the last decade, there has been a heightened interest in adjunctive interventions for depressive disorders. In particular, incorporating physical exercise (PE) into pharmacological and psychological interventions has been shown to further reduce depressive symptoms, enhance overall health, and lower relapse rates [8,9,10,11,12]. PE offers several benefits, including affordability, ease of access, self-administration, and a favorable side effect profile, which confers additional health advantages for patients afflicted with depression.

The majority of the scientific literature indicates that both aerobic and resistance training modalities are effective in reducing depressive symptoms [13,14]. Furthermore, certain mind–body practices, such as Tai Chi and yoga, have been demonstrated to provide substantial benefits for this condition [15,16], notwithstanding WHO guidelines recommending 75–150 min of vigorous or 150–300 min of moderate physical activity weekly [17]. Moreover, supervised exercise programs, compared with unsupervised ones, improve engagement and retention, especially among individuals with MDD [18,19], which could lead to better outcomes in reducing depression symptoms. While several meta-analyses have clearly demonstrated that exercise is a viable and well-tolerated adjunct therapy [20,21], uncertainty persists regarding the optimal exercise dose; specifically, the appropriate duration, intensity, and frequency to achieve significant and sustained reductions in depressive symptoms. Many studies investigating short- and medium-term effects lack follow-up data [10,22,23]. Furthermore, each patient faces distinct challenges, particularly those experiencing severe symptoms of depression.

According to the PubMed database, the number of meta-analyses (147) of randomized controlled trials (RCTs) assessing the effects of exercise on depressive disorder has steadily increased since 2010. Among these, 22 studies focused on individuals without additional comorbidities; however, none conducted meta-regressions or closely monitored physical activity. Therefore, the main goal of this systematic review with meta-analysis and meta-regression is to thoroughly evaluate the effectiveness of conventional PE as an additional treatment for adults diagnosed with depressive disorder. It also aims to improve clinical practice and promote the integration of exercise into standard care protocols.

## 2. Results

### 2.1. Search Results

**Figure 1:**
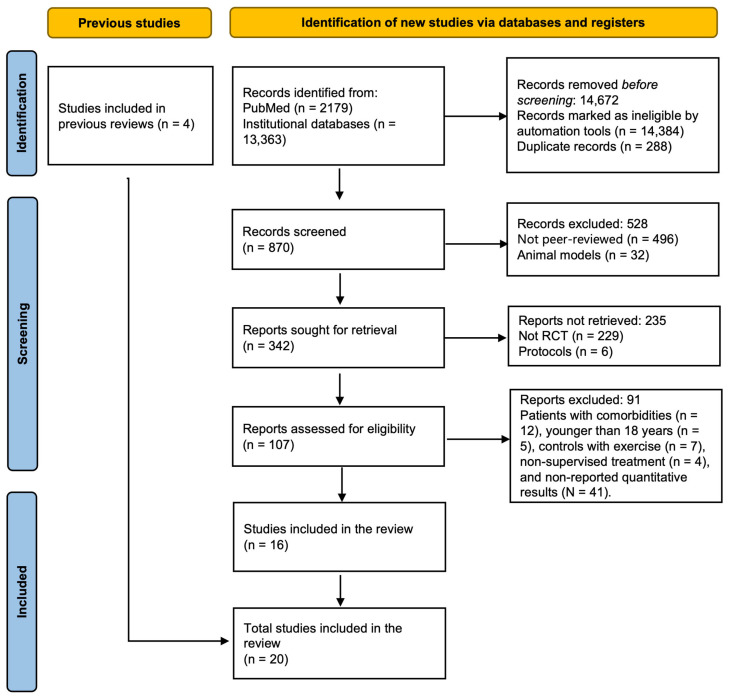
Flowchart of the study selection.

In the initial phase of our search strategy, a total of 15,542 studies were identified, comprising 2179 from PubMed and 13,363 from institutional databases. Following automated screening, 14,384 studies were excluded, of which 288 were identified as duplicates. Of the 870 potentially pertinent articles identified, 496 were not peer-reviewed, and 32 involved animal models. Among the remaining 342 articles, 229 were not randomized controlled trials (RCTs), and 6 were protocols. During the full-text review process, 107 articles were examined, of which 91 were excluded for the following reasons: patients with comorbidities (*n* = 12), participants younger than 18 years (*n* = 5), control trials with exercise (*n* = 7), non-supervised exercise treatment (*n* = 4), and non-reported quantitative results (*n* = 41) (Figure 1). Ultimately, 20 full texts met the predefined eligibility criteria, including four from previous reviews.

### 2.2. Characteristics of Participants

Across the selected 20 studies, a total of 1202 adults diagnosed with depression were included: 334 males and 868 females, with ages ranging from 18 to 70 years. In three studies [24,25,26], participants exhibited low to moderate depressive symptoms (*n* = 175), whereas the remaining subjects had MDD (*n* = 1027). In two studies [24,26], the participants did not report taking antidepressants (*n* = 133); the others (*n* = 1069) were using antidepressant medications, including SSRIs, lithium, tricyclics, among others.

### 2.3. Characteristics of the Studies

Studies on patients with low depression levels show low heterogeneity (I^2^ 8–25%), while those with high depression levels exhibit moderate heterogeneity (59–75%). Overall heterogeneity ranges from 61% to 76%. In 3 studies with 7 interventions, patients had low depression levels, whereas 17 studies with 19 interventions showed high depression levels. Twelve interventions used the BDI-II, 6 used the HAM-D, 5 used the HADS, 1 used the MADRS-S, and 1 used the DASS-21.

### 2.4. Characteristics of Interventions

The exercise interventions included aerobic training (15 interventions), multimodal exercise (4 interventions), Tai Chi (3 interventions), Yoga (2 interventions), resistance training (1 intervention), and stretching (1 intervention) (Table 1). The activities in the control group included light stretching and rest during the waiting period. The duration of treatment varied from 10 days to 16 weeks. Exercise intensity levels ranged from low to vigorous, with the total number of sessions ranging from 12 to 60.

### 2.5. Risk of Bias (See Section 4.6)

**Figure 2:**
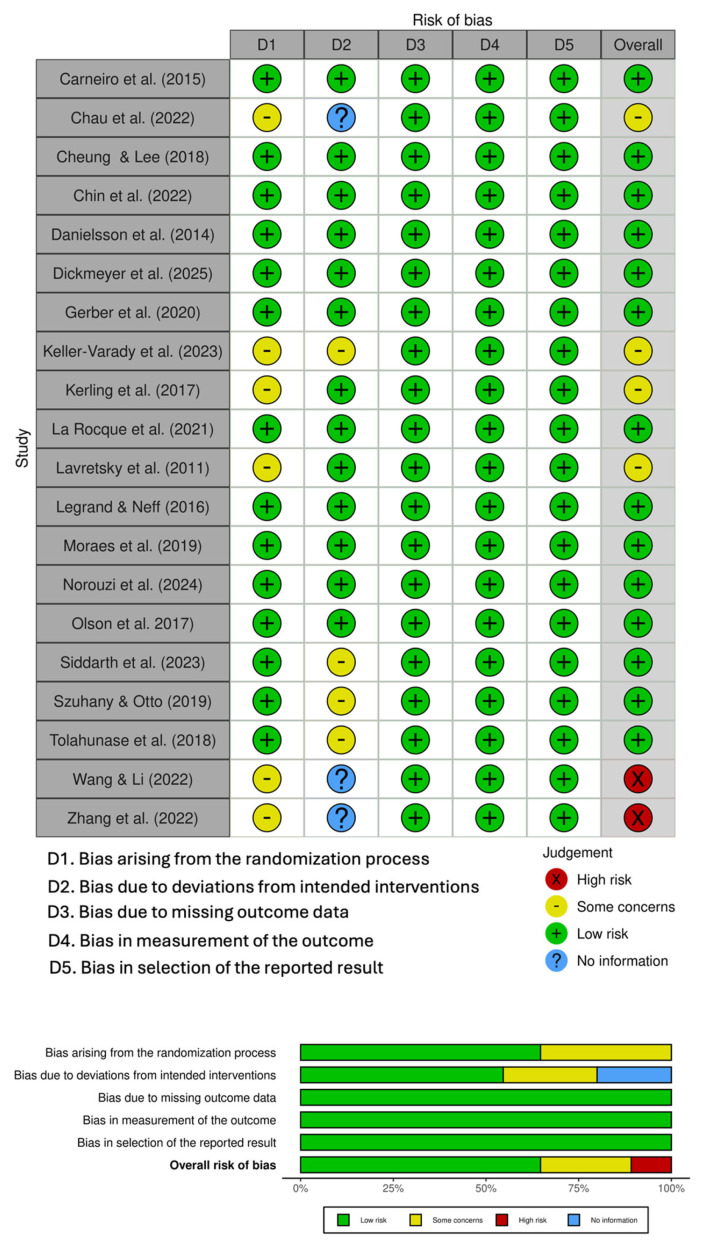
Risk of bias of the selected manuscripts. References [10,14,23,24,25,26,27,28,29,30,31,32,33,34,35,36,37,38,39,40] are cited in this figure.

**Figure 3:**
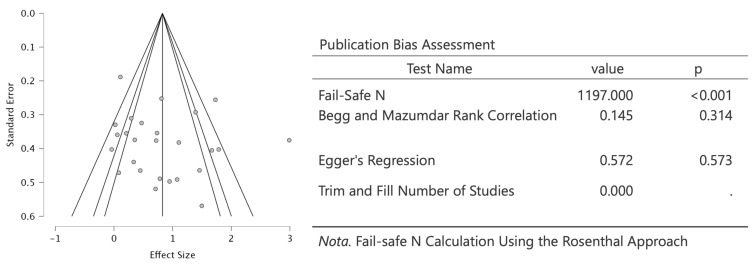
Funnel plot and tests of publication bias.

Overall, 14 studies were assessed as having good methodological rigor and a low risk of bias; 4 studies exhibited some concerns; and two studies were identified as having a high risk of bias. Three studies did not provide information regarding the intended interventions (Figure 2). The overall risk of bias was classified as follows: 70% low risk, 20% with some concerns, and 10% with high risk (Figure 2). The funnel plot, the Begg and Mazumdar test, and Egger’s regression test indicated the absence of publication bias (Figure 3).

### 2.6. Meta-Regression

From the 20 included studies, 52 groups (treatment and control cohorts) were analyzed. The meta-regression analysis demonstrates that the instrument employed to assess depression severity and total work performed (workload) accounts for 74% of the variance in the pooled effect size (Table 2).

**Figure 4:**
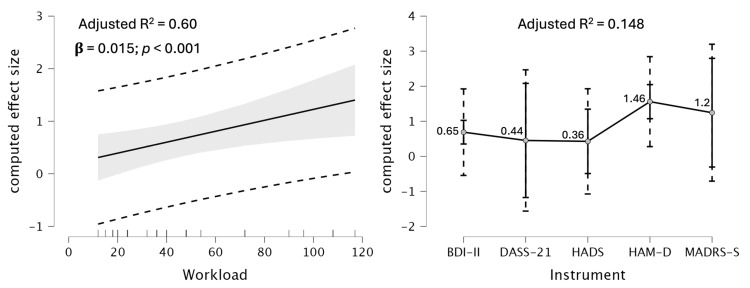
Marginal Effects Plots on computed effect size. Shading = confidence intervals, dashed lines = prediction intervals.

Additionally, among the moderators (Table 3 and Figure 4), the influence of workload (F = 9.8; *p* = 0.005; adjusted R^2^ = 0.60) was larger than that of instrument type (F = 3.5; *p* = 0.025; adjusted R^2^ = 0.148). The unstandardized coefficients for BDI-II, HAM-D, and MADRS-S were positive (0.189, 1.063, 693, respectively), while DASS-21 and HADS were negative (−0.1, −0.108, respectively).

### 2.7. Meta-Analysis

**Figure 5:**
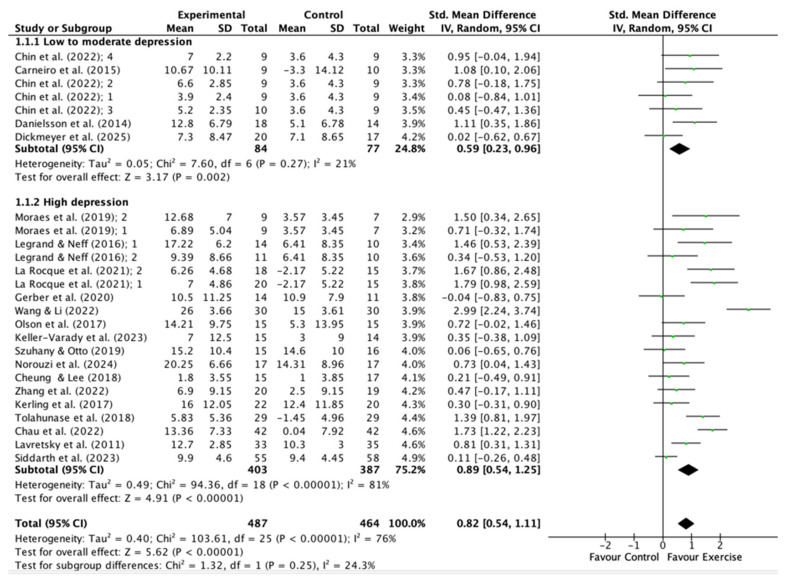
Forest plot of the effect of physical exercise on depression symptoms in individuals with depressive disorder: Classical meta-analysis, without moderators. References [10,14,23,24,25,26,27,28,29,30,31,32,33,34,35,36,37,38,39,40] are cited in this figure.

**Figure 6:**
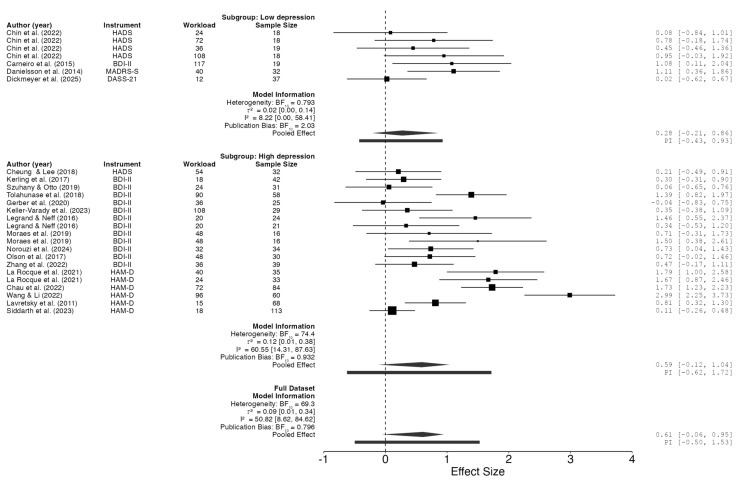
Forest plot of the effect of physical exercise on depression symptoms in individuals with depressive disorder: Robust Bayesian Meta-Analysis with workload and type of instrument as moderators. Workload in arbitrary units. References [10,14,23,24,25,26,27,28,29,30,31,32,33,34,35,36,37,38,39,40] are cited in this figure.

The analysis, excluding moderating factors, indicated that the SMD ranged from −0.039 to 2.989, with most estimates (96%) being positive (Figure 5). The estimated average SMD, derived from the random-effects model, was 0.82 (95% CI: 0.54–1.11). This suggests that the mean outcome differed significantly from zero (t(19) = 5.62, *p* < 0.0001). The Q-test results indicate heterogeneity among the true effect sizes (Q(19) = 103.61, *p* < 0.0001, tau^2^ = 0.40, I^2^ = 76%). The 95% prediction interval for the true effects ranges from −0.49 to 2.15. Given the considerable heterogeneity across studies and the influence of confounding variables, specifically, instrument and workload, these two factors were incorporated as moderators in both the classical and the Robust Bayesian Meta-analysis. The classical meta-analysis yielded consistent findings (SMD = 0.80, 95% CI: 0.57 to 1.03; I^2^ = 59%) and a 95% prediction interval (PI) ranging from −0.15 to 1.74, while the Robust Bayesian Meta-analysis (RoBMA R Code; Supplementary File S1) provided a conservative estimate of the pooled effect size (SMD = 0.61, 95% CI: −0.06 to 0.95; PI: from −0.50 to 1.53; I^2^ = 51%) (Figure 6). Thus, although the average outcome is estimated to be positive, some studies may yield negative results. Analyzing the studentized residuals and Cook’s distances, one study [39] had a value exceeding ±3.10 and could be considered a potential outlier exerting substantial influence. Then, in the sensitivity analysis, excluding this study reduced the SMD in both classical and robust Bayesian meta-analysis to 0.71 (95% CI: 0.49 to 0.93; PI: −0.12 to 1.53; I^2^ = 52%) and 0.49 (95% CI: −0.09 to 0.88; PI: −0.60 to 1.40; I^2^ = 47%), respectively. Neither the rank correlation nor the regression test indicated any asymmetry (*p* = 0.31 and *p* = 0.57, respectively (Figure 3).

## 3. Discussion

The present systematic review and meta-analysis update offers a rigorous and comprehensive evaluation of the antidepressant effects of supervised physical exercise in individuals diagnosed with depressive disorder. The pooled analysis indicates a clinically and statistically significant reduction in depressive symptoms among participants who engage in supervised physical exercise interventions (SMD = 0.82, CI: 0.54–1.25; SMD = 0.61, CI: −0.06–0.95, in classical and robust Bayesian meta-analysis, respectively). According to Cohen’s conventions [43], this effect size is considered high, underscoring the clinical significance of exercise as an intervention modality for depressive disorder. Nevertheless, the analysis also identified low to moderate heterogeneity among the studies included (I^2^ = 8–76%), reflecting substantial variability in intervention characteristics, participant populations, and study methodologies. However, when confounding variables are included in the model (depression level, workload, and the instrument used to evaluate depression), the pooled effect size remains high (SMD = 0.80) and heterogeneity decreases considerably (I^2^ = 51%) (Supplementary File S1). Additionally, using a robust Bayesian meta-analysis, the pooled effect size, although moderate (SMD = 0.61), was clinically significant: only 4% of studies favored the control group, highlighting the positive impact of exercise as an adjunct intervention to conventional treatments in reducing depressive symptoms.

The comprehensive search strategy, strict adherence to PRISMA guidelines, and rigorous risk-of-bias assessment using the Cochrane Collaboration tool validate the strength and relevance of our findings. Additionally, the observed high pooled effect size is particularly meaningful given the heterogeneity of trials, exercise modalities, exercise intensities, and participant characteristics. This supports the role of exercise as an effective, accessible, and cost-efficient intervention for treating depression [44].

A comprehensive comparison with earlier systematic reviews and meta-analyses strengthens the consistency of the antidepressant effect of physical exercise. For example, Schuch et al. (2016) reported a high effect size (SMD = 0.99, 95% CI: 0.69–1.29) across 33 randomized clinical trials involving 1877 participants in resistance exercise protocols [12]. Cooney & Mead (2013) observed a moderate effect size (SMD = −0.62, 95% CI: −0.81 to −0.42, I^2^ = 63%) in their Cochrane review across 35 RCTs involving 1353 participants, but a lower effect size (SMD = 0.45, 95% CI: 0.06–0.83, I^2^ = 33%) in 4 methodologically robust trials [44]. Gordon et al. reported a moderate effect size (SMD = 0.66, 95% CI: 0.48 to 0.83, I^2^ = 76%) in 33 RCTs involving 1877 participants [45]. Kvam et al. (2016) reported a moderate effect (SMD = −0.68, 95% CI: −0.92 to −0.44, I^2^ = 68%) in 23 RCTs involving 977 participants [46]. Nevertheless, a meta-review reported a nonsignificant pooled effect size (SMD = 0.11, 95% CI: −0.41–0.18) in 4 high-quality trials [41], suggesting limited clinical efficacy. A recent network meta-analysis involving 14,170 participants across 218 studies observed moderate to low reductions in depression. Among these, walking/jogging demonstrated the highest effect size (SMD = 0.63, 95% CI: 46–80), followed by yoga (SMD = −0.55, 95% CI: 36–73), strength training (SMD = 0.49, 95% CI: 29–69), and cycling, which exhibited the lowest effect (SMD = 0.30, 95% CI: 0.01–60), with the observed effects proportionate to the specific exercise modalities’ intensity [42].

Unlike the literature, the pooled effect size in this study is moderately high, which may reflect careful control over inclusion criteria and the studied populations (without added comorbidities). In addition, the notable heterogeneity identified in this review is not unique; earlier meta-analyses have similarly documented substantial between-study variability, often attributed to differences in exercise modality, intensity, duration, supervision, and participant characteristics [12,44,47].

This work did not identify similar studies that employed moderators alongside rigorous Bayesian methods. Nevertheless, if the research objective were not solely to determine fixed parameters (means, standard deviations, *p*-values, etc.), we advocate using both analyses, owing to their greater robustness, flexibility, and extensive applicability in the health sector [11,48].

The novelty of this study lies in integrating statistically significant moderator variables into the model, alongside a comparative analysis of classical and Bayesian meta-analyses of the pooled effect sizes, methodologies that have traditionally received limited scrutiny. This amalgamation of statistical techniques enabled the isolation of moderator effects on the overall effect size and facilitated the calculation of its true value, thereby enhancing the reliability and clinical applicability of the findings. Additionally, it was observed that larger workloads are associated with larger effect sizes.

The overall effect size of the two moderators is also considered high (Adjusted R^2^ = 0.744) [49], with workload serving as the primary moderator (Adjusted R^2^ = 0.612), in contrast to the instrument (Adjusted R^2^ = 0.132); effect sizes were not documented in previous studies. This represents a pioneering discovery that warrants consideration in future research exploring the impact of exercise on depression. Although earlier research has established the efficacy of exercise, few studies have systematically investigated the sources of heterogeneity arising from variations in physical activity and participants’ depression levels [12,44]. By addressing these gaps, this review offers a more detailed understanding of how, for whom, and under what conditions exercise is most effective.

Additionally, the ongoing global burden of MDD, the limitations of pharmacological and psychotherapeutic options, and the urgent need for affordable and sustainable intervention solutions underscore the importance of this study. In this sense, exercise, as demonstrated here, provides a compelling complement or alternative to conventional therapies, with a favorable side effect profile and additional health benefits, including reduced cardiovascular risk and improved metabolic function [50]. The evidence further supports the notion that engaging in physical exercise may help prevent depressive symptoms, irrespective of age or other health conditions [51,52].

From a clinical perspective, the incorporation of exercise into treatment protocols for depressive disorder is supported by the consistency and magnitude of numerous positive outcomes, as well as by international directives from the World Health Organization [1,53] and the National Institute for Health and Care Excellence [54]. Both organizations endorse physical activity as either a primary or complementary intervention for depression. Given the accessibility and scalability of exercise interventions, they are particularly valuable in settings with limited resources, among populations with restricted access to mental health services, and for patients with different levels of depression. Moreover, unlike antidepressant medications, which may be associated with a range of adverse effects and adherence difficulties, exercise is typically well tolerated, thereby promoting overall health and improving quality of life. Nevertheless, the significant heterogeneity identified in the current analysis underscores the need for further investigation, particularly regarding the adoption of a standardized methodology for assessing depression, participants’ depression levels, and, most importantly, the regulation of exercise type and number of sessions and intensities.

A complex interplay of neurobiological and psychological mechanisms supports the antidepressant effects of exercise in individuals with depression. From a neurobiological perspective, aerobic exercise improves neurocognitive function [23] and can increase volume in specific brain regions such as the amygdala, thalamus, and nucleus accumbens with low to moderate effect size (0.26 to 0.54) [55], fostering neuroplasticity and hippocampal neurogenesis, and modulates monoaminergic neurotransmission (serotonin, dopamine, norepinephrine); all of these processes contribute to mitigating the pathology of depression [50,56,57].

Exercise also has anti-inflammatory effects, lowering levels of pro-inflammatory cytokines such as IL-6 and TNF-α, which are elevated in depression [8,57], helping to normalize hypothalamic–pituitary–adrenal axis activity and reducing hyperactivity and stress [8,14]. The social benefits are increased communication, social support, and reduced loneliness [58,59], especially when exercise occurs in natural environments (green exercise) [60]. Psychologically, physical exercise boosts energy, motivation, self-concept, self-efficacy, resilience, and social connections, and provides a sense of mastery and achievement, all of which contribute to improvements in mental health and mood, as well as a reduction in depressive symptoms [22,61,62].

A key strength of the present review is its focus on moderating factors that impact the effectiveness of exercise interventions for depression. In addition to this work, the existing literature provides substantial evidence that the following factors are crucial in influencing treatment outcomes.

- Type of Exercise: Aerobic exercise (e.g., walking, jogging, cycling) and resistance training are the most extensively studied modalities, with both demonstrating large effect sizes [42,63]. In contrast, yoga and mind–body exercises show moderate effects, especially in older adults and individuals with comorbidities [42,47].
- Intensity: The antidepressant effects of exercise are proportional to the intensity prescribed, with moderate to vigorous exercise yielding the most significant benefits [42,47,63]. However, even light physical activity confers clinically meaningful effects, especially in previously inactive individuals [42].
- Duration and Frequency: Interventions lasting 6–12 weeks, with sessions of 30–60 min and performed 3–4 times per week, are associated with optimal outcomes [20,63]. Short interventions may also produce large effects, possibly attributable to greater participant adherence to the programs and the novelty of the activities; however, sustained engagement is necessary for long-term benefits [42,47].
- Supervision and Group Format: Supervised and group-based interventions are more effective than unsupervised or individual formats, likely due to increased motivation, accountability, and social support [42,64].
- Participant Characteristics: Age, sex, baseline depression severity, and comorbidities may also influence the response to exercise; however, the evidence remains inconclusive. Certain studies indicate that women might derive greater benefits from strength training, whereas older adults tend to respond favorably to yoga and walking [12,47,52].

Despite its strengths, this review possesses certain limitations. Primarily, due to the limited number of selected manuscripts, it includes only two moderators from the extensive range mentioned earlier (such as sex, age, type of exercise, and exercise supervision). Secondly, the moderate to high heterogeneity observed (I^2^ = 51–76%) limits the accuracy of the pooled effect estimate, suggesting that the antidepressant effects of exercise may differ significantly across populations and intervention protocols. Thirdly, the presence of potential outliers, as in Wang & Li (2022), may further introduce bias into the outcomes [39]. Fourthly, reliance on published studies raises the possibility of publication bias, though robust statistical techniques and sensitivity analyses can mitigate this concern to some extent. Therefore, incorporating moderating variables into models, performing sensitivity analyses, and employing robust statistical methods, such as Bayesian meta-analysis, are useful.

However, methodological diversity across studies creates significant heterogeneity, affecting the accuracy of results. In this regard, differences in the outcome measures and diagnostic criteria used to evaluate depression across studies may affect the comparability of our results. Additionally, the absence of other moderators, such as aphaty and abulia, along with the lack of long-term follow-up data in the included studies, also hinders definitive conclusions about the long-term effects of exercise-related improvements in depressive symptoms.

Another limitation is the variability in outcome measures and diagnostic criteria used to evaluate depression across studies, which may impact the comparability of results. The lack of long-term follow-up data in many included studies also prevents definitive conclusions about the sustainability of exercise-related improvements in depressive symptoms. The review’s emphasis on RCTs and controlled studies may reduce its relevance to real-world settings, where adherence to exercise interventions is often lower, and comorbidities are more common. Future research should include pragmatic trials and implementation studies to better assess the effectiveness of exercise in routine clinical practice.

## 4. Materials and Methods

### 4.1. Registration

This study followed the procedures of the Preferred Reporting Items for Systematic Reviews and Meta-analysis (PRISMA) 2020 statement [65] (Supplementary File S2). Previously, the protocol was registered in the International Prospective Register of Systematic Reviews (PROSPERO) database (registration number: CRD420251121919). The methodological criteria outlined in this work are provided in Table 4.

### 4.2. Search Strategy

A comprehensive, systematic, and computerized search was conducted across PubMed/MEDLINE and two institutional multidatabase platforms (The National Autonomous University of Mexico and the Autonomous University of Ciudad Juárez), which included the following databases: EBSCOhost, Ovid, Web of Science, and Scopus. The PICOS (population, intervention, comparison, outcome, and study design) was used as the search methodology (Table 5). Literature published from 1 January 2010, to 31 August 2025, was incorporated, employing the keywords ((exercis* OR aerobic* OR running OR jogging OR walk* OR hiking OR swim* OR aquatic* OR cycling OR bicycl* OR strength*) AND (depressi*) AND ((randomized clinical trial) OR (randomized controlled trial)), adapted for each database. The following filters were applied to the databases: RCTs, articles in English or Spanish, availability of an abstract, publication date after 2010, and inclusion of human participants aged 18 years or older.

### 4.3. Eligibility Criteria

Only primary original research of randomized controlled trials (RCTs) and recent reviews that aimed to objectively study depression through any kind of physical exercise-based interventions in patients with depression were included. Exercise was defined as a planned, structured, and repetitive intervention aimed at improving or maintaining physical conditioning. Eligible reports involved (a) adult individuals (age ≥ 18) with diagnostic criteria for depression or MDD established by validated instruments, such as The Diagnostic and Statistical Manual of Mental Disorders (DSM)-IV, DSM-IV-TR, DSM-5 [66,67], International Classification of Diseases (ICD)-10, or ICD-11 [68], and confirmed through a validated structured diagnostic interview such as the Beck Depression Inventory (BDI) [69], Hamilton Depression Rating Scale (HAM-D) [70], Patient Health Questionnaire (PHQ-9) [71], Center for Epidemiologic Studies Depression Scale (CES-D) [72], Montgomery-Åsberg Depression Rating Scale (MADRS) [70], Zung Self-Rating Depression Scale (Zung SDS) [73], The Geriatric Depression Scale (GDS) [74], Depression Anxiety Stress Scales (DASS) [75], or Hospital Anxiety and Depression Scale (HADS) [76]; (b) studies published or accepted for publication; (c) comparisons focused on a passive physical activity, non-exercise, or wait-list control group, with a focus on individuals with depression; and (d) supervised exercise. Manuscripts with non-randomized trials, meeting abstracts, meta-analyses, reviews, and case reports, as well as studies involving individuals with comorbidities such as chronic degenerative diseases (e.g., hypertension, cancer, diabetes, sarcopenia), severe psychiatric disorders, and those in which medication was altered during the study were excluded.

### 4.4. Study Selection

A compilation of potentially pertinent studies was assembled for subsequent review and screening. Two independent reviewers assessed each article’s eligibility based on its title and abstract (A.R-J. and R.P.H-T.). The references and abstracts from the reviewed articles were imported into Zotero for reference management, where duplicates were eliminated. Subsequently, the screening and manuscript selection were performed by two authors, who examined the full texts and reached consensus on the inclusion of articles in the final list. A third reviewer was engaged to resolve any uncertainties concerning the inclusion or exclusion of studies. The methodology for assessing eligibility conforms to the criteria delineated in the Cochrane Handbook for Systematic Reviews of Interventions, version 6.5 [77], and adheres to the PRISMA guidelines [65] for article reporting (Figure 1). Corresponding authors were contacted when study abstracts met the inclusion criteria, but the full texts were inaccessible, or when trials lacked essential information for meta-analytical procedures.

### 4.5. Data Extraction

From each article included in the review, relevant data were extracted, such as sex, age, diagnostic criteria, inpatient/outpatient status, number of participants, year of publication, treatment type, duration, exercise frequency, intensity, workload, depression severity, and the instrument used to determine depression level (Table 1). For each trial, we also recorded the sample sizes, means, and standard deviations (SDs) reported for each outcome (Supplementary File S3). We use Excel (version 16.104) and Zotero (version 7.0.29) to record and organize data. Three authors (A.R.-J., R.P.H.-T., and I.A.C.-G.) worked independently during data collection. When SDs were unavailable, we estimated them using precision measures such as standard errors, 95% confidence intervals, or *p*-values.

### 4.6. Risk of Bias Assessment

The risk-of-bias assessment of the included trials was conducted independently by two reviewers (R.P.H-T., M.T-T) following the Cochrane Collaboration’s methods [64]. A third reviewer (I.A.C-G) was involved to resolve discrepancies between the initial assessments. The following criteria were evaluated: (1) bias stemming from the randomization process; (2) bias due to deviations from the intended interventions; (3) bias resulting from missing outcome data; (4) bias in the measurement of outcomes; and (5) bias in the selection of the reported results. The classification was based on the categories: “low risk,” “some concerns,” “high risk,” or “no information.” To generate risk-of-bias visualizations, the Revised Cochrane Risk of Bias RoB 2 tool for randomized trials was used to assess methodological quality and internal validity [77]. The visual figures were produced using the web platform robvis (https://mcguinlu.shinyapps.io/robvis/, accessed on 17 December 2025) [78].

### 4.7. Statistical Analysis

The analysis was conducted employing the standardized mean difference (SMD) as the primary outcome measure. Initially, to assess the influence of moderating factors on the pooled effect, a meta-regression was performed with effect size as the dependent variable. The instruments used to assess depressive disorder and workload were the independent variables. Each study measured exercise intensity using different tools, methods, and units (Supplementary File S3); therefore, for this study, exercise intensity was categorized into arbitrary units. So, the workload was calculated by multiplying the exercise intensity (classified as low = 1, moderate = 2, and high = 3) by the number of sessions completed by participants. Given their predictive role and the limited number of selected randomized controlled trials (RCTs), these two independent variables were incorporated into the meta-analytic model. The severity of depression was analyzed separately. A random-effects model was fitted to the data. Heterogeneity was quantified through the estimate of tau squared (τ^2^), obtained via the restricted maximum likelihood estimator. Additionally, the Q-test for heterogeneity and the I-squared (I^2^) statistic were reported. In cases where heterogeneity was detected (i.e., τ^2^ > 0 regardless of the Q-test outcomes), a prediction interval for the true effect sizes was provided. Additionally, to ensure the accuracy and convergence of the Bayesian model, we set the autofit R-hat to 1.01 and the effective sample size to 500, given the observed high heterogeneity and outliers (See the RoBMA R code, Supplementary File S1). Statistical tests and confidence intervals were computed using the Knapp-Hartung method. Studentized residuals and Cook’s distances were used to identify potential outliers and influential studies within the model. Studies with a studentized residual exceeding the 100 × (1 − 0.05/(2 × k)) percentile of the standard normal distribution were considered potential outliers, and a Bonferroni correction was applied with a two-sided alpha level of 0.05 for k studies included in the meta-analysis. Studies with a Cook’s distance exceeding the median plus six times the interquartile range of Cook’s distances were deemed influential. A sensitivity analysis was conducted by removing the study with the highest Cook’s distance. Publication bias was assessed using the Begg and Mazumdar rank correlation test and Egger’s regression test, with the standard error of the observed outcomes as the predictor, as visualized in the asymmetry funnel plot. The meta-analysis without moderators was performed utilizing RevMan version 5.4.1 [79]. The moderator effects were incorporated into the Classical Meta-Analysis and the Robust Bayesian Meta-Analysis, utilizing the JASP software (Version 0.95.3) [80].

## 5. Conclusions

In summary, this updated systematic review with meta-analysis and meta-regression supports the evidence that supervised physical exercise provides a moderate, clinically meaningful antidepressant effect and should be considered as a complement or alternative to traditional therapies for individuals with depressive disorders, especially those who are pharmacotherapy resistant. Additionally, we found that exercise frequency and intensity are important determinants of exercise efficacy, with higher workload associated with a greater antidepressant effect (R^2^ = 0.60). Furthermore, this factor should be considered when interpreting results, given the variety of standardized tools used to assess depression. Therefore, our results indicate the need to increase both the session frequency and exercise intensity in protocols to reduce depression.

Since exercise is usually well-tolerated and accessible, it is a valuable option in settings with limited resources, among populations with restricted access to mental health services, and useful for patients with varying levels of depression. In addition, considering the global burden of depression and the limitations inherent in current treatments, integrating exercise into standard care protocols is a vital health strategy priority.

## Figures and Tables

**Table 1:** General characteristics of the 20 chosen manuscripts.

Author (Year)	Sample Size (Treatment/Control)	Age, y	Gender M/F	Instrument	Grade of Depression	Drugs	Intervention (Treatment/Control)	Time of Treatment	Number of Sessions	Exercise Intensity
Carneiro et al. (2015) [27]	9/10	18–65	0/19	BDI-II	Low to moderate	Yes	Aerobic exercise/rest	16 weeks	39	65–80% MHR
Chau et al. (2022) [28]	42/42	18–64	17/67	HAM-D	High	Yes	Multimodal exercise/rest	12 weeks	36	50–70% MHR
Cheung & Lee (2018) [29]	15/17	18–65	7/34	HADS	High	Yes	Aerobic exercise/rest	12 weeks	36	60% MHR
Chin et al. (2022) [24]	9/9	≥60	3/18	HADS	Low to moderate	NM	Aerobic exercise/light stretching	12 weeks	12	∼3.25 METs
Chin et al. (2022) [24]	9/9	≥60	3/18	HADS	Low to moderate	NM	Aerobic exercise/light stretching	12 weeks	36	∼3.25 METs
Chin et al. (2022) [24]	10/9	≥60	4/19	HADS	Low to moderate	NM	Aerobic exercise/light stretching	12 weeks	12	∼6.5 METs
Chin et al. (2022) [24]	9/9	≥60	3/18	HADS	Low to moderate	NM	Aerobic exercise/light stretching	12 weeks	36	∼6.5 METs
Danielsson et al. (2014) [25]	18/14	18–65	10/32	MADRS-S	Low to moderate	Yes	Aerobic exercise/rest	10 weeks	20	Moderate
Dickmeyer et al. (2025) [26]	20/17	18–70	37/0	DASS-21	Low to moderate	NM	Aerobic exercise/rest	6 weeks	6	3.5 METs
Gerber et al. (2020) [14]	14/11	18–61	0/25	BDI-II	High	Yes	Aerobic exercise/rest	6 weeks	18	60–75% MHR
Keller-Varady et al. (2023) [30]	15/14	18–60	4/27	BDI-II	High	Yes	Multimodal exercise/rest	6 weeks	36	Moderate-to-vigorous
Kerling et al. (2017) [31]	22/20	18–60	26/16	BDI-II	High	Yes	Aerobic exercise/rest	6 weeks	18	50% MHR
La Rocque et al. (2021) [32]	20/15	18–65	0/35	HAM-D	High	Yes	Multimodal exercise/rest	8 weeks	16	Moderate
La Rocque et al. (2021) [32]	18/15	18–65	0/33	HAM-D	High	Yes	Yoga/rest	8 weeks	16	Low to moderate
Lavretsky et al. (2011) [33]	33/35	≥60	26/42	HAM-D	High	Yes	Tai Chi/rest	10 weeks	10	Low to moderate
Legrand & Neff (2016) [10]	14/10	27–67	8/16	BDI-II	High	Yes	Aerobic exercise/rest	10 days	10	65–75% MHR
Legrand & Neff (2016) [10]	11/10	27–67	8/17	BDI-II	High	Yes	Stretching/rest	10 days	10	65–75% MHR
Moraes et al. (2019) [34]	9/7	≥60	3/13	BDI-II	High	Yes	Aerobic exercise/rest	12 weeks	24	70% MHR
Moraes et al. (2019) [34]	9/7	≥60	3/13	BDI-II	High	Yes	Resistance training/rest	12 weeks	24	70% 1-MR
Norouzi et al. (2024) [35]	17/17	18–70	8/26	BDI-II	High	Yes	Multimodal exercise/rest	8 weeks	16	70% MHR
Olson et al. (2017) [23]	15/15	18–30	6/24	BDI-II	High	Yes	Aerobic exercise/light stretching	8 weeks	24	40–65% HR reserve
Siddarth et al. (2023) [36]	55/58	≥60	31/82	HAM-D	High	Yes	Tai Chi/rest	12 weeks	12	Low to moderate
Szuhany & Otto (2019) [37]	15/16	18–65	5/16	BDI-II	High	Yes	Aerobic exercise/light stretching	12 weeks	12	Moderate
Tolahunase et al. (2018) [38]	29/29	19–50	27/31	BDI-II	High	Yes	Yoga/rest	12 weeks	60	Low to moderate
Wang & Li (2022) [39]	30/30	NM	NM	HAM-D	High	Yes	Aerobic exercise/rest	8 weeks	32	120–150 beats/min
Zhang et al. (2022) [40]	20/19	30–60	4/35	BDI-II	High	Yes	Tai Chi/rest	12 weeks	24	Low to moderate

Beck Depression Inventory (BDI) [26], Hamilton Depression Rating Scale (HAM-D) [39], Montgomery-Åsberg Depression Rating Scale (MADRS) [39], Depression Anxiety Stress Scales (DASS) [41], or Hospital Anxiety and Depression Scale (HADS) [42], Heart Rate (HR), Maximal Heart Rate (MHR), Maximal Repetition (MR), Metabolic Equivalent of Task (MET).

**Table 2:** ANOVA table of Meta-regression.

Model		Sum of Squares	df	Mean Square	F	*p*-Value	R^2^ Adjusted
M_1_	Regression	19.056	1	19.056	39.432	<0.001	0.612
	Residual	12.082	25	0.483			
	Total	31.138	26				
M_2_	Regression	25.011	6	4.169	13.608	<0.001	0.744
	Residual	6.127	20	0.306			
	Total	31.138	26				

Note. M_1_ includes Workload; M_2_ includes Workload and Instrument. Degrees of freedom (df).

**Table 3:** Effect Size Meta-Regression Terms Tests.

	Subgroup	F	df_1_	df_2_	*p*-Value
Workload	Full dataset	9.759	1	20	0.005
	Low depression	15.823	1	2	0.058
	High depression	6.582	1	15	0.022
Instrument	Full dataset	3.515	4	20	0.025
	Low depression	10.211	3	2	0.091
	High depression	4.385	2	15	0.032

Note. Fixed effects tested using Knapp and Hartung adjustment. Degrees of freedom (df).

**Table 4:** Resume of methodological criteria covered in this work.

Quality Characteristics	Covered in Meta-Analysis?	Details
Protocol pre-registration	Yes	PROSPERO (CRD420251121919)
Use of PRISMA	Yes	PRISMA 2020 reporting
Independent screening and data extraction	Yes	Two independent reviewers; third reviewer for consensus
Comprehensive search	Yes	Multiple databases, PICOS strategy
Standardized eligibility criteria	Yes	DSM/ICD diagnosis, validated instruments, exclusion of comorbidities/med changes
Use of validated depression assessment tools	Yes	BDI, HAM-D, PHQ-9, CES-D, MADRS, etc.
Risk of bias (RoB 2) assessment	Yes	All five domains, two blinded reviewers
Sensitivity/subgroup analyses	Yes	Performed as part of results
Transparent study flow and exclusion reporting	Yes	PRISMA diagram, full text review and exclusion reasons
Handling of missing data	Yes	SDs estimated from SEs, CIs, *p*-values if needed
Statistical rigor (heterogeneity, influence, funnel)	Yes	Tau^2^, Q-test, I^2^, Cook’s D, studentized residuals, funnel plot asymmetry
Assessment of comorbidity	Yes	Included as a section in results
Blinding of outcome assessors	Yes (as RoB domain)	Not always feasible for exercise; assessed as risk of bias domain
Adverse event/safety reporting	Not explicitly	Not detailed in the provided text
Power analysis/sample size in included studies	Yes	In meta-analysis description
Long-term follow-up	Not systematically	Mentioned as a gap, not systematically analyzed

**Table 5:** PICOS search methodology.

Population	Adults (age ≥ 18) with depressive disorder
Intervention	Physical exercise
Comparison	Individuals in passive physical activity, non-exercise, or wait-list control group.
Outcome	Depression, or MDD diagnosed using validated instruments, sample size, age, sex, depression severity, treatment type, treatment duration, and exercise intensity
Study design	Systematic review with meta-analysis and meta-regression of randomized controlled trials

## Data Availability

The original contributions presented in this study are included in the article/Supplementary Material. Further inquiries can be directed to the corresponding author.

## Supplementary Materials

The following supporting information can be downloaded at: https://www.mdpi.com/article/10.3390/jfmk11010009/s1.
