# Supplementary material for: An Update on the Effect of Physical Exercise on Depressive Disorder: A Systematic Review with Meta-Analysis and Meta-Regression of Randomized Controlled Trials
Source: J Funct Morphol Kinesiol. 2025 Dec 25;11(1):9. doi: 10.3390/jfmk11010009 (PMC12821582; doi:10.3390/jfmk11010009)
Supplement: Supplementary file 1 [file jfmk-11-00009-s001.zip › Supplementary file S1.pdf]

Forest Plot of Classical Meta-Analysis

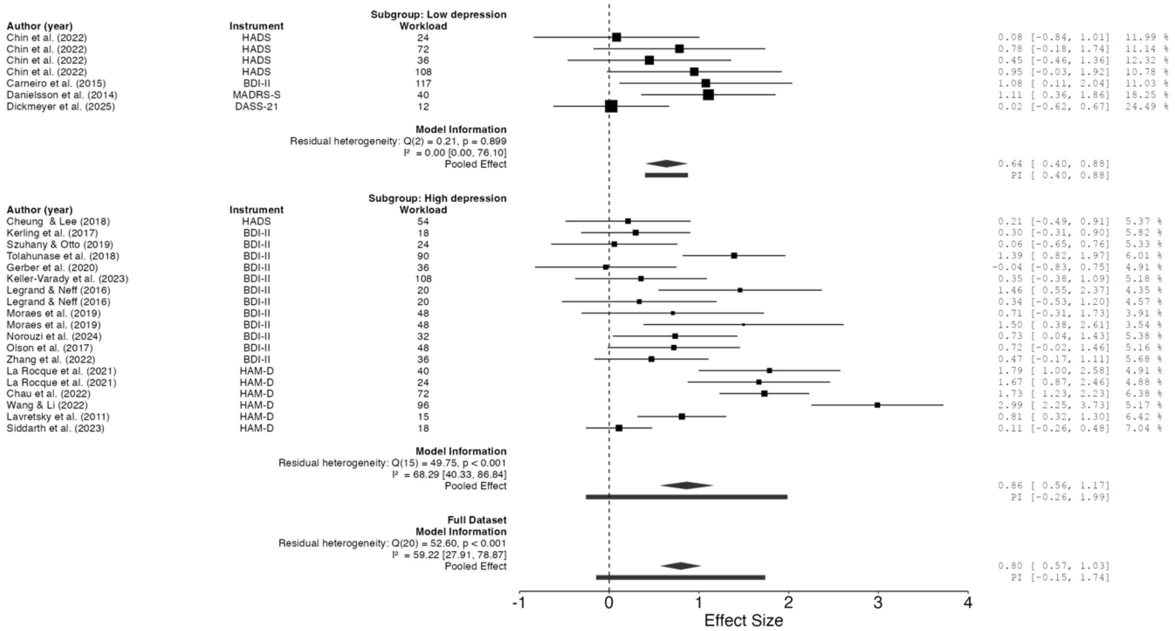

Forest Plot of Robust Bayesian Meta-Analysis

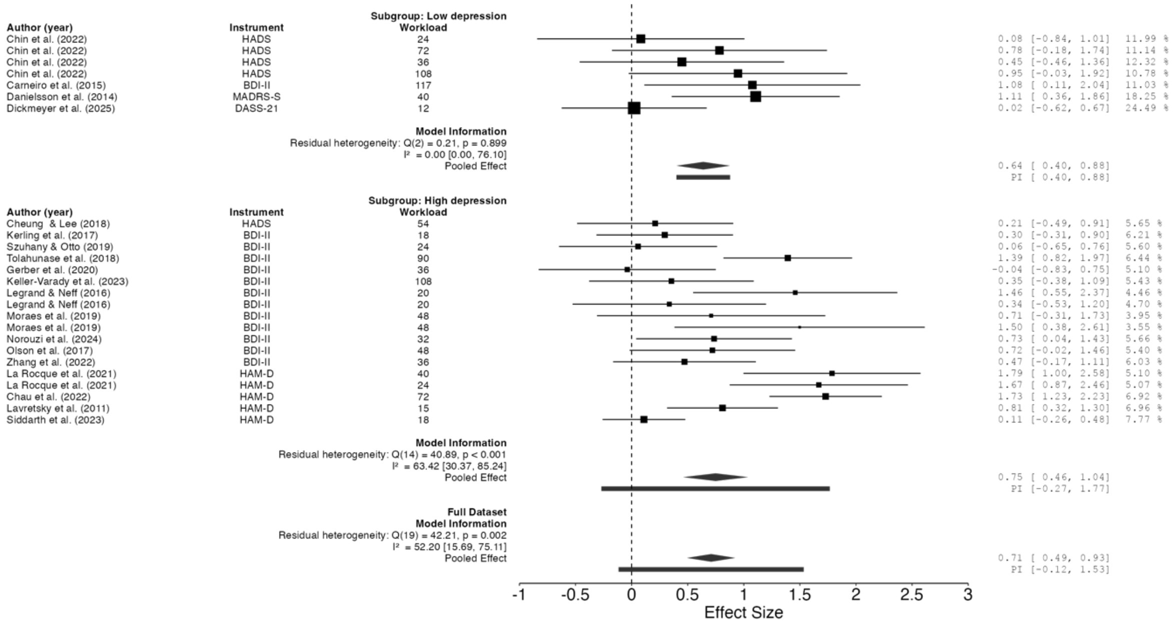

Sensitivity analysis: Forest Plot of Robust Bayesian Meta-Analysis

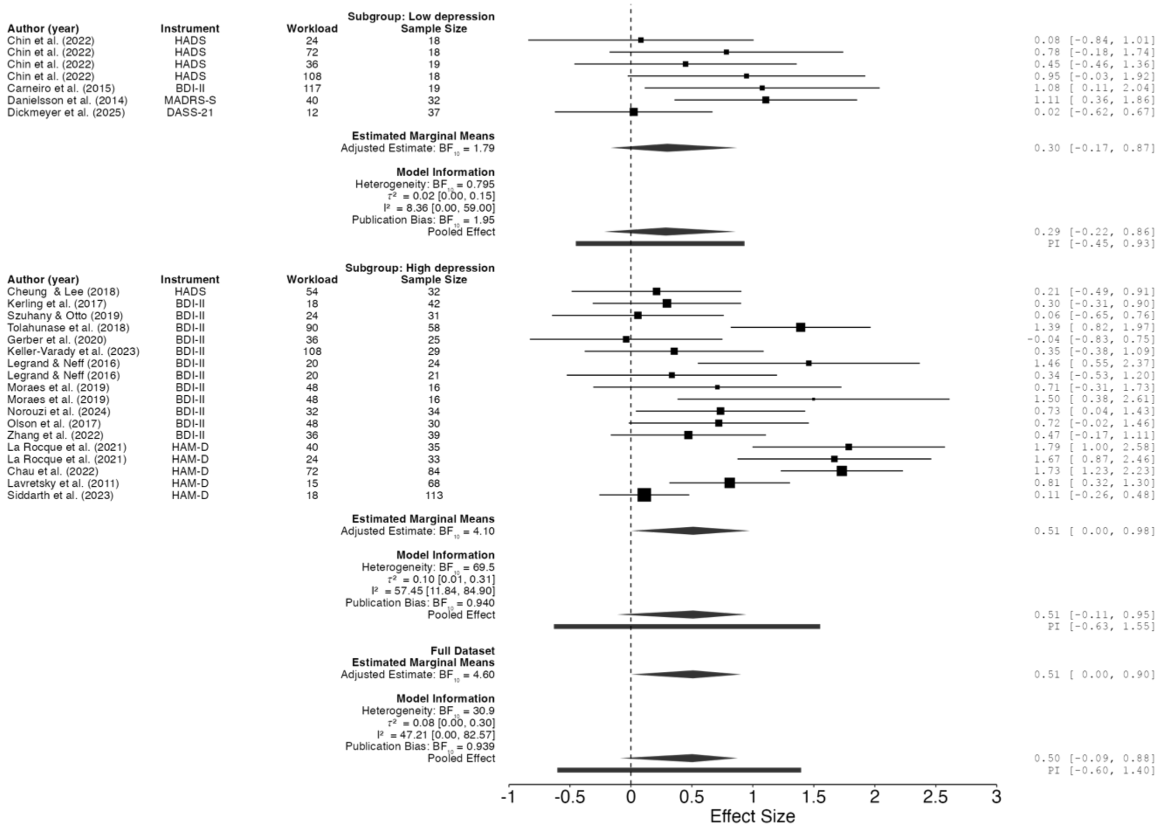

RoBMA R Code

```r
colnames(dataset)[colnames(dataset) == 'computed effect size'] <- 'd'
colnames(dataset)[colnames(dataset) == 'computed standard error'] <- 'se'

fit <- RoBMA.reg(
        formula = ~Workload + Instrument,
        data = dataset,
        priors = list(
                Workload = list(
                        alt = prior(distribution = 'normal', parameters = list(mean = 0, sd = 0.25), truncation = list(lower = -Inf, upper = Inf), prior_weights = 1),
                        null = prior(distribution = 'point', parameters = list(location = 0), prior_weights = 1)
                ),
                Instrument = list(
                        alt = prior_factor(distribution = 'mnormal', parameters = list(mean = 0, sd = 0.25), truncation = list(lower = -Inf, upper = Inf), contrast = 'meandif', prior_weights = 1),
                        null = prior_factor(distribution = 'mpoint', parameters = list(location = 0), contrast = 'meandif', prior_weights = 1)
                )
        ),
        prior_scale = 'cohens_d',
        transformation = 'fishers_z',
        priors_effect = list(
                prior(distribution = 'normal', parameters = list(mean = 0, sd = 1), truncation = list(lower = -Inf, upper = Inf), prior_weights = 1)
        ),
        priors_heterogeneity = list(
                prior(distribution = 'invgamma', parameters = list(shape = 1, scale = 0.15), truncation = list(lower = 0, upper = Inf), prior_weights = 1)
        ),
        priors_effect_null = list(
                prior(distribution = 'point', parameters = list(location = 0), prior_weights = 1)
        ),
        priors_heterogeneity_null = list(
                prior(distribution = 'point', parameters = list(location = 0), prior_weights = 1)
        ),
        priors_bias = list(
                prior_weightfunction(distribution = 'two.sided', parameters = list(steps = 0.05, alpha = c(1, 1)), prior_weights = 0.0833333333333333),
                prior_weightfunction(distribution = 'two.sided', parameters = list(steps = c(0.05, 0.1), alpha = c(1, 1, 1)), prior_weights = 0.0833333333333333),
                prior_weightfunction(distribution = 'one.sided', parameters = list(steps = 0.05, alpha = c(1, 1)), prior_weights = 0.0833333333333333),
                prior_weightfunction(distribution = 'one.sided', parameters = list(steps = c(0.025, 0.05), alpha = c(1, 1, 1)), prior_weights = 0.0833333333333333),
                prior_weightfunction(distribution = 'one.sided', parameters = list(steps = c(0.05, 0.5), alpha = c(1, 1, 1)), prior_weights = 0.0833333333333333),
                prior_weightfunction(distribution = 'one.sided', parameters = list(steps = c(0.025, 0.05, 0.5), alpha = c(1, 1, 1, 1)), prior_weights = 0.0833333333333333),
                prior_PET(distribution = 't', parameters = list(location = 0, scale = 1, df = 1), truncation = list(lower = 0, upper = Inf), prior_weights = 0.25),
                prior_PEESE(distribution = 't', parameters = list(location = 0, scale = 5, df = 1), truncation = list(lower = 0, upper = Inf), prior_weights = 0.25)
        ),
        priors_bias_null = list(
                prior_none(prior_weights = 1)
        ),
        effect_direction = 'positive',
        chains = 3,
        adapt = 4500,
        burnin = 6500,
        sample = 14000,
        thin = 1,
        autofit = TRUE,
        autofit_control = list(max_Rhat = 1.01, min_ESS = 500, max_error = NULL, max_SD_error = NULL, max_time = NULL, sample_extend = 5000, restarts = 10, max_extend = 10),
        algorithm = 'ss'
)
```
