# Supplementary material for: An Update on the Effect of Physical Exercise on Depressive Disorder: A Systematic Review with Meta-Analysis and Meta-Regression of Randomized Controlled Trials
Source: J Funct Morphol Kinesiol. 2025 Dec 25;11(1):9. doi: 10.3390/jfmk11010009 (PMC12821582; doi:10.3390/jfmk11010009)
Supplement: Supplementary file 1 [file jfmk-11-00009-s001.zip › Supplementary file S2 PRISMA_2020_checklist.pdf]

PRISMA

PRISMA 2020 Checklist

Section and Topic	Item #	Checklist item	Location where item is reported
**TITLE**			
Title	1	An update on the effect of physical exercise on depressive disorder: A systematic review with meta-analysis and meta-regression of randomized controlled trials	Lines 2-4
**ABSTRACT**			
Abstract	2	Background: Physical exercise (PE) has emerged as a promising intervention for de-pressive disorder (DD), yet its efficacy and optimal implementation remain under investigation. Objective: To thoroughly assess the effectiveness of structured PE as a conventional treatment for adults with DD. Methods: Adhering to PRISMA 2020 guidelines, a comprehensive literature search was conducted across PubMed/MEDLINE and institutional multi-databases. Peer-reviewed randomized controlled trials (RCTs) published between 2010 and 2025 that studied adults diagnosed with DD without other comorbidities under supervised exercise interventions were selected. Methodological rigor (search, screening, and manuscript selection) was ensured through two independent reviewers. The influence of moderating variables (workload and the instrument used to evaluate DD: instrument) was analyzed using meta-regression, and the pooled effect size was estimated using both frequentist and Bayesian meta-analyses. Results: From 15,542 screened records, 20 RCTs met the inclusion criteria. Workload and instrument account for 60% and 15% of the variance in the effect size. Supervised PE significantly reduced depressive symptoms (standardized mean difference = 0.80; 95% CI: 0.57–1.03; I^2 = 59%). The robust Bayesian meta-analysis confirmed the consistency of these findings. Conclusion: PE is a valuable complementary therapy that significantly reduces depressive symptoms in adults with depression. PROSPERO registration number: CRD420251121919	Lines 19-36
**INTRODUCTION**			
Rationale	3	Depressive disorder represents a significant challenge to global public health, affecting millions of individuals worldwide and imposing considerable societal and per-sonal burdens [1]. With an estimated prevalence of 332 million people globally, major depressive disorder (MDD) is a primary cause of disability, with projections indicating an even greater future burden. Data from 2021 in the United States indicate that approximately 21.0 million adults (8.3%) experienced at least one episode of MDD, with a higher prevalence among females (10.3%) than among males (6.3%) [3]. Symptoms encompass persistent low mood, anhedonia, fatigue, low self-esteem, and hopelessness [1]. Moreover, comorbidities such as substance abuse, suicidal ideation, and chronic pain are also prevalent [4]. These conditions diminish quality of life and impose substantial economic costs due to decreased productivity. Depression is not merely a mental health disorder but also a major risk factor for numerous adverse health outcomes, including higher mortality rates, chronic diseases, disability, and poor prognosis in related conditions [5]. Furthermore, a complex interplay between biological, psychological, and social factors elevates the risk of depression, including sleep disturbances, trauma, low social support, and socioeconomic disadvantages [6].<br><br>The pursuit of effective, accessible, and sustainable treatments for depressive dis-order is thus an urgent priority. Especially, numerous patients with MDD do not achieve remission or discontinue treatment due to various factors, including illness severity, comorbidities, high patient expectations, and adverse medication side effects [7]. Consequently, over the last decade, there has been a heightened interest in adjunctive therapies for depressive disorder. In particular, physical exercise (PE) has considerable evidence supporting its effectiveness as an adjunctive treatment for depressive disorder, serving as an alternative to traditional therapies and first-line antidepressants, while enhancing overall health and reducing relapse rates [8–10]. PE offers several benefits, including affordability, ease of access, self-administration, and a favorable side effect profile, which confers additional health advantages for patients afflicted with depression.<br><br>The majority of the scientific literature indicates that both aerobic and resistance training modalities are effective in reducing depressive symptoms [11,12]. Furthermore, certain mind-body practices, such as Tai Chi and yoga, have been demonstrated to provide substantial benefits for this condition [13,14], notwithstanding WHO guidelines recommending 75–150 minutes of vigorous or 150–300 minutes of moderate physical activity weekly [15]. Moreover, supervised exercise programs, compared with unsupervised ones, improve engagement and retention, especially among individuals with MDD [16,17], which could lead to better outcomes in reducing depression symptoms. While several meta-analyses have clearly demonstrated that exercise is a viable and well-tolerated adjunct therapy [18,19], uncertainty persists regarding the optimal exercise dose; specifically, the appropriate duration, intensity, and frequency to achieve significant and sustained reductions in depressive symptoms. Many studies investigating short- and medium-term effects lack follow-up data [10,20,21]. Furthermore, each patient faces distinct challenges, particularly those experiencing severe symptoms of depression.<br><br>According to the PubMed database, the number of meta-analyses (147) of randomized controlled trials (RCTs) assessing the effects of exercise	Lines 43-92

PRISMA

PRISMA 2020 Checklist

Section and Topic	Item #	Checklist item	Location where item is reported
		on depressive disorder has steadily increased since 2010. Among these, 22 studies focused on individuals without additional comorbidities; however, none conducted meta-regressions or closely monitored physical activity	
Objectives	4	Evaluate the effectiveness of structured physical exercise as an additional treatment for adults diagnosed with depressive disorder. It also aims to improve clinical practice and promote the integration of exercise into standard care protocols.	Lines 88-92
**METHODS**			
Eligibility criteria	5	Only original, peer-reviewed randomized controlled trials (RCTs) and recent re-views that aimed to objectively study depression through any kind of physical exercise-based interventions in patients with depression were included. Exercise was defined as a planned, structured, and repetitive intervention aimed at improving or maintaining physical conditioning. Eligible reports involved (a) adult individuals (age ≥ 18) with diagnostic criteria for depression or MDD established by validated instruments, such as The Diagnostic and Statistical Manual of Mental Disorders (DSM)-IV, DSM-IV-TR, DSM-5 [23,24], International Classification of Diseases (ICD)-10, or ICD-11 [25] and confirmed through a validated structured diagnostic interview such as the Beck Depression Inventory (BDI) [26], Hamilton Depression Rating Scale (HAM-D) [27], Patient Health Questionnaire (PHQ-9) [28], Center for Epidemiologic Studies Depression Scale (CES-D) [29], Montgomery-Åsberg Depression Rating Scale (MADRS) [27], Zung Self-Rating Depression Scale (Zung SDS) [30], The Geriatric Depression Scale (GDS) [31], Depression Anxiety Stress Scales (DASS)[32], or Hospital Anxiety and Depression Scale (HADS) [33]. (b) Peer-reviewed studies published or accepted; (c) Comparisons focused on a passive physical activity, non-exercise, or wait-list control group, with a focus on individuals with depression; (d) supervised exercise. Manuscripts with non-randomized trials, meeting abstracts, meta-analyses, reviews, case reports, as well as studies involving individuals with comorbidities such as chronic degenerative diseases associated (e.g., hypertension, cancer, diabetes, sarcopenia), severe psychiatric disorders, and those in which medication was altered during the study were excluded.	Lines 374-396
Information sources	6	A comprehensive, systematic, and computerized search was conducted across PubMed/MEDLINE and the institutional multi-database platforms of the National Autonomous University of Mexico and the Autonomous University of Ciudad Juárez, which included EBSCOhost, Ovid, Web of Science, and Scopus, utilizing the PICOS (population, intervention, comparison, outcome, and study design) search methodology (Table 5). Literature published from January 1, 2010, to August 31, 2025, was incorporated, employing	Lines 360-372
Search strategy	7	Keywords ((exercis* OR aerobic* OR running OR jogging OR walk* OR hiking OR swim* OR aquatic* OR cycling OR bicycl* OR strength*) AND (depressi*) AND ((randomized clinical trial) OR (randomized controlled trial)), adapted for each database. The following filters were applied in the databases: RCTs, articles in English, Spanish, or Portuguese; availability of an abstract; publication date subsequent to 2010; and inclusion of human participants aged 18 years or older.	Lines 360-372
Selection process	8	A compilation of potentially pertinent studies was assembled for subsequent review and screening. Two independent reviewers evaluated each article's eligibility by examining its title and abstract. The references from the reviewed articles were imported into Zotero for reference management, where duplicates were systematically eliminated. Subsequently, the two authors examined the full texts and reached a consensus regarding the inclusion of articles in the final list. A third reviewer was engaged to resolve any uncertainties concerning the inclusion or exclusion of studies. The methodology for assessing eligibility conforms to the criteria delineated in the Cochrane Handbook for Systematic Reviews of Interventions, version 6.5 [34], and adheres to the PRISMA guidelines [22] for article reporting (Figure 1). Corresponding authors were contacted when study abstracts met the inclusion criteria, but the full texts were inaccessible, or when trials lacked essential information for meta-analytical procedures	Lines 397-410
Data collection process	9	From each article included in the review, relevant data were extracted, such as sex, age, diagnostic criteria, inpatient/outpatient status, number of participants, year of publication, treatment type, duration, exercise frequency, intensity, workload, depression severity, and the instrument used to determine depression level (Table 1). For each trial, we also recorded the sample sizes, means, and standard deviations (SDs) reported for each outcome (supplementary file 1). We use Excel and Zotero software to record and organize the data. Three authors (A.R-J, R.P.H-T and I.A.C-G) worked independently in the data collection process. When SDs were unavailable, we estimated them using measures of precision such as standard errors, 95% confidence intervals, or p-values	Lines 411-420
Data items	10a	Depression, or MDD diagnosed using validated instruments, sample size, age, sex, depression severity, treatment type, treatment duration, and exercise intensity.	Lines 372-373. Table 5.

PRISMA 2020 Checklist

Section and Topic	Item #	Checklist item	Location where item is reported
	10b	The Diagnostic and Statistical Manual of Mental Disorders (DSM)-IV, DSM-IV-TR, DSM-5 [23,24], International Classification of Diseases (ICD)-10, or ICD-11 [25] and confirmed through a validated structured diagnostic interview such as the Beck Depression In-ventory (BDI) [26], Hamilton Depression Rating Scale (HAM-D) [27], Patient Health Questionnaire (PHQ-9) [28], Center for Epidemiologic Studies Depression Scale (CES-D) [29], Montgomery-Åsberg Depression Rating Scale (MADRS) [27], Zung Self-Rating Depression Scale (Zung SDS) [30], The Geriatric Depression Scale (GDS) [31], Depression Anxiety Stress Scales (DASS)[32], or Hospital Anxiety and Depression Scale (HADS) [33]. (b) Peer-reviewed studies published or accepted; (c) Comparisons focused on a passive physical activity, non-exercise, or wait-list control group, with a focus on individuals with depression; (d) supervised exercise. Manuscripts with non-randomized trials, meeting abstracts, meta-analyses, reviews, case reports, as well as studies involving individuals with comorbidities such as chronic degenerative diseases associated (e.g., hypertension, cancer, diabetes, sarcopenia), severe psychiatric disorders, and those in which medication was altered during the study were excluded.<br>Corresponding authors were contacted when study abstracts met the inclusion criteria, but the full texts were inaccessible, or when trials lacked essential information for meta-analytical procedures.<br>When SDs were unavailable, we estimated them using measures of precision such as standard errors, 95% confidence intervals, or p-values.	Lines 380-396, 408-410, 419-420.
Study risk of bias assessment	11	The risk of bias assessment in the included trials was conducted independently by two reviewers (R.P.H-T., M.T-T), following the Cochrane Collaboration's methods [34]. A third reviewer (I.A.C-G) was involved to resolve discrepancies between the initial assessments. The following criteria were evaluated: (1) bias stemming from the randomization process; (2) bias due to deviations from the intended interventions; (3) bias resulting from missing outcome data; (4) bias in the measurement of outcomes; and (5) bias in the selection of the reported results. The classification was based on the categories: "low risk," "some concerns," "high risk," or "no information." To generate risk-of-bias visualizations, the Revised Cochrane Risk of Bias RoB 2 tool for randomized trials was used to assess methodological quality and internal validity [34]). The visual figures were produced using the web platform robvis (https://mcguinlu.shinyapps.io/robvis/) [35.	Lines 421-432
Effect measures	12	For each trial, we also recorded the sample sizes, means, and standard deviations (SDs) reported for each outcome (supplementary file 1). We use Excel and Zotero software to record and organize the data. Three authors (A.R-J, R.P.H-T and I.A.C-G) worked in-dependently in the data collection process. When SDs were unavailable, we estimated them using measures of precision such as standard errors, 95% confidence intervals, or p-values.	Lines 414-420
Synthesis methods	13a	A compilation of potentially pertinent studies was assembled for subsequent review and screening. Two independent reviewers evaluated each article's eligibility by examining its title and abstract. The references from the reviewed articles were imported into Zotero for reference management, where duplicates were systematically eliminated. Subsequently, the two authors examined the full texts and reached a consensus regarding the inclusion of articles in the final list. A third reviewer was engaged to resolve any uncertainties concerning the inclusion or exclusion of studies. The methodology for assessing eligibility conforms to the criteria delineated in the Cochrane Handbook for Systematic Reviews of Interventions, version 6.5 [34], and adheres to the PRISMA guidelines [22] for article reporting (Figure 1). Corresponding authors were contacted when study abstracts met the inclusion criteria, but the full texts were inaccessible, or when trials lacked essential information for meta-analytical procedures.<br>The methodology for assessing eligibility conforms to the criteria delineated in the Cochrane Handbook for Systematic Reviews of Interventions, version 6.5 [34], and adheres to the PRISMA guidelines [22] for article reporting (Figure 1). Corresponding authors were contacted when study abstracts met the inclusion criteria, but the full texts were inaccessible, or when trials lacked essential information for meta-analytical procedures	Lines 397-410, 433-466
	13b	The analysis was conducted employing the standardized mean difference (SMD) as the primary outcome measure. Initially, to assess the influence of moderating factors on the pooled effect, a meta-regression was performed with effect size as the dependent variable. The instruments employed to evaluate depressive disorder and workload served as the independent variables. Workload was calculated by multiplying the exercise intensity (classified as low = 1, moderate = 2, and high = 3) by the number of sessions completed by participants. Given their role as predictors and the limited number of selected randomized controlled trials (RCTs), these two independent variables were incorporated into the meta-analytic model. The severity of depression was analyzed separately. A random-effects model was fitted to the data. Heterogeneity was quantified through the estimate of tau squared (τ^2), obtained via the restricted maximum likelihood estimator. Additionally, the Q-test for heterogeneity and the I-squared (I^2) statistic were reported. In instances where heterogeneity was detected (i.e., τ^2> 0 regardless of the	Lines 433-466

Section and Topic	Item #	Checklist item	Location where item is reported
		Q-test outcomes), a prediction interval for the true effect sizes was provided. Statistical tests and confidence intervals were computed using the Knapp-Hartung method. Studentized residuals and Cook's distances were employed to identify potential outliers and influential studies within the model. Studies with a studentized residual exceeding the 100 × (1 - 0.05/(2 × k)) percentile of the standard normal distribution were considered potential outliers, and a Bonferroni correction was applied with a two-sided alpha level of 0.05 for k studies included in the meta-analysis. Studies with a Cook's distance exceeding the median plus six times the interquartile range of Cook's distances were deemed influential. A sensitivity analysis was conducted by removing the study with the highest Cook's distance. Publication bias was assessed using the Begg and Mazumdar rank correlation test and Egger's regression test, with the standard error of the observed outcomes as the predictor, as visualized in the asymmetry funnel plot. The meta-analysis without moderators was performed utilizing RevMan version 5.4.1 [36]. The moderator effects were incorporated into the Classical Meta-Analysis and the Robust Bayesian Meta-Analysis, utilizing the JASP software [37]	
	13c	The references and abstracts from the reviewed articles were imported into Zotero for reference management, where duplicates were systematically eliminated.<br>We use Excel and Zotero software to record and organize the data.<br>When SDs were unavailable, we estimated them using measures of precision such as standard errors, 95% confidence intervals, or p-values.	Lines 400-402, 417-419.
	13d	The analysis was conducted employing the standardized mean difference (SMD) as the primary outcome measure. Initially, to assess the influence of moderating factors on the pooled effect, a meta-regression was performed with effect size as the dependent variable…	Lines 434-437
	13e	Heterogeneity was quantified through the estimate of tau squared (τ^2), obtained via the restricted maximum likelihood estimator. Additionally, the Q-test for heterogeneity and the I-squared (I^2) statistic were reported. In instances where heterogeneity was detected (i.e., $\tau^2 > 0$ regardless of the Q-test outcomes), a prediction interval for the true effect sizes was provided	Lines 446-453
	13f	Studies with a studentized residual exceeding the 100 × (1 - 0.05/(2 × k)) percentile of the standard normal distribution were considered potential outliers, and a Bonferroni correction was applied with a two-sided alpha level of 0.05 for k studies included in the meta-analysis. Studies with a Cook's distance exceeding the median plus six times the interquartile range of Cook's distances were deemed influential. A sensitivity analysis was conducted by removing the study with the highest Cook's distance.	Lines 455-460
Reporting bias assessment	14	Publication bias was assessed using the Begg and Mazumdar rank correlation test and Egger's regression test, with the standard error of the observed outcomes as the predictor, as visualized in the asymmetry funnel plot.	Lines 461-463
Certainty assessment	15	A random-effects model was fitted to the data. Heterogeneity was quantified through the estimate of tau squared (τ^2), obtained via the restricted maximum likelihood estimator…	Lines 445-450
**RESULTS**			
Study selection	16a	In the initial phase of our search strategy, a total of 15,542 studies were identified, comprising 2,179 from PubMed and 13,363 from institutional databases. Following automated screening, 14,384 studies were excluded, of which 288 were identified as duplicates. Of the 870 potentially pertinent articles identified, 496 were not peer-reviewed, and 32 involved animal models. Among the remaining 342 articles, 229 were not randomized controlled trials (RCTs), and 6 were protocols. During the full-text review process, 107 articles were examined, of which 91 were excluded for various reasons (Figure 1). Ultimately, 20 full texts met the predefined eligibility criteria, including four from previous reviews.	Lines 95-105
	16b	Explained in Figure 2	Line 106
Study characteristics	17	Across the selected 20 studies, a total of 1,202 adults diagnosed with depression were included: 334 males and 868 females, with ages ranging from 18 to 70 years. In three studies [38–40], participants exhibited low to moderate depressive symptoms (n=175), whereas the remaining subjects had MDD (n=1,027). In two studies [38,40], the participants did not report taking antidepressants (n=133); the others (n=1,069) were using antidepressant medications, including SSRIs, lithium, tricyclics, among others. The exercise interventions included aerobic training, multimodal exercise, resistance training, Yoga, and Tai Chi (Table 1). The activities in the control group included light stretching and rest during the waiting period. The duration of treatment varied from 10 days to 16 weeks. Exercise intensity levels ranged from low to vigorous, with the total number of sessions ranging from 12 to 60.	Lines 108-114.<br>Table 1, line 129
Risk of bias in	18	Overall, 14 studies were assessed as having good methodological rigor and a low risk of bias; 4 studies exhibited some concerns; and two	Lines

PRISMA 2020 Checklist

Section and Topic	Item #	Checklist item	Location where item is reported
studies		studies were identified as having a high risk of bias. Three studies did not provide information regarding the intended interventions (Figure 2). The overall risk of bias was classified as follows: 70% low risk, 20% with some concerns, and 10% with high risk (Figure 2). The funnel plot, the Begg and Mazumdar test, and Egger's regression test indicated the absence of publication bias (Figure 3).	130137. Figure 2
Results of individual studies	19	Table 1	Lines 129
Results of syntheses	20a	Risk of vias<br>Figure 2	Lines 139
	20b	Meta-regression<br>Meta-analysis<br>Tables 2,3<br>Figures 4-6.	Lines 132-145,147-174.
	20c	Meta-analysis	Lines 179-188
	20d	Then, in the sensitivity analysis, excluding this study reduced the SMD to 0.71 (95% CI: 0.49 to 0.93; PI: -0.12 to 1.53; I^2 = 52%). Neither the rank correlation nor the regression test indicated any asymmetry (p = 0.31 and p = 0.57, respectively (Figure 3).	Lines 174-177
Reporting biases	21	The funnel plot, the Begg and Mazumdar test, and Egger's regression test indicated the absence of publication bias (Figure 3).	Lines 134-137
Certainty of evidence	22	The overall risk of bias was classified as follows: 70% low risk, 20% with some concerns, and 10% with high risk.<br>Risk of bias of the selected manuscripts (Figure 2)	Lines 134-137,138-139
**DISCUSSION**			
Discussion	23a	The present systematic review and meta-analysis offer a rigorous and comprehensive evaluation of the antidepressant effects of physical exercise in individuals diagnosed with depressive disorder. The pooled analysis indicates a clinically and statistically significant reduction in depressive symptoms among participants who engage in structured physical exercise interventions (SMD = 0.82, CI: 0.54-1.25). According to Cohen's conventions [42], this effect size is considered high, underscoring the clinical significance of exercise as a therapeutic modality for depressive disorder. Nevertheless, the analysis also identified considerable heterogeneity among the studies included (I^2 = 76%), reflecting substantial variability in intervention characteristics, participant populations, and study method-ologies. However, when confounding variables are included in the model (depression level, workload, and the instrument used to evaluate depression), the effect size remains high (SMD = 0.80) and heterogeneity decreases considerably (I^2 = 59%). Additionally, using robust Bayesian meta-analysis, the pooled effect size, although moderate (SMD = 0.61), was clinically significant, as only 4% of studies favored the control group, highlighting the positive impact of exercise as an adjunct therapy to conventional treatments in reducing depression symptoms	Lines 189-195
	23b	Nevertheless, the significant heterogeneity identified in the current analysis underscores the need for further investigation, particularly regarding the adoption of a standardized methodology for assessing depression, participants' depression levels, and, most importantly, the regulation of exercise type and workload (number of sessions and intensities).	Lines 197-199,
	23c	Despite its strengths, this review possesses certain limitations. Primarily, due to the limited number of selected manuscripts, it includes only two moderators from the ex-tensive range mentioned earlier (such as sex, age, type of exercise, and exercise super-vision). Secondly, moderate to high heterogeneity (I^2 approximately 51-76%) limits the accuracy of the pooled effect estimate, suggesting that the antidepressant effects of exercise may differ significantly across populations and intervention protocols. Thirdly, the presence of potential outliers, as in the study by Wang & Li (2022), may further introduce bias into the outcomes [41]. Fourthly, reliance on published studies raises the possibility of publication bias, though robust statistical techniques and sensitivity analyses can mitigate this concern to some extent. Therefore, the usefulness of incorporating	Lines 324-335

PRISMA

PRISMA 2020 Checklist

Section and Topic	Item #	Checklist item	Location where item is reported
		moderating variables into the models, performing sensitivity analysis, and using robust statistical analyses, such as Bayesian meta-analysis.<br>Another limitation is the variability in outcome measures and diagnostic criteria used to evaluate depression across studies, which may impact the comparability of results. The lack of long-term follow-up data in many included studies also prevents definitive conclusions about the sustainability of exercise-related improvements in depressive symptoms. The review's emphasis on RCTs and controlled studies may reduce its relevance to real-world settings, where adherence to exercise interventions is often lower and comorbidities are more common. Future research should include pragmatic trials and implementation studies to better assess the effectiveness of exercise in routine clinical practice	
	23d	Therefore, the usefulness of incorporating moderating variables into the models, per-forming sensitivity analysis, and using robust statistical analyses, such as Bayesian meta-analysis.<br>Another limitation is the variability in outcome measures and diagnostic criteria used to evaluate depression across studies, which may impact the comparability of results. The lack of long-term follow-up data in many included studies also prevents definitive conclusions about the sustainability of exercise-related improvements in depressive symptoms.	Lines 333-335
**OTHER INFORMATION**			
Registration and protocol	24a	PROSPERO registration number: CRD420251121919.	Lines 35, 357, 359
	24b	PROSPERO registration number: CRD420251121919.	Lines 35, 357, 359
	24c	NA	
Support	25	NA	
Competing interests	26	The authors declare no conflicts of interest of review authors.	Lines 494-496
Availability of data, code and other materials	27	The APC was funded by PRODEP UACH and UABC..	Line 489

From: Page MJ, McKenzie JE, Bossuyt PM, Boutron I, Hoffmann TC, Mulrow CD, et al. The PRISMA 2020 statement: an updated guideline for reporting systematic reviews. BMJ 2021;372:n71. doi: 10.1136/bmj.n71.
This work is licensed under CC BY 4.0. To view a copy of this license, visit https://creativecommons.org/licenses/by/4.0/
